# 1.15 Å resolution structure of the proteasome-assembly chaperone Nas2 PDZ domain

**DOI:** 10.1107/S2053230X14003884

**Published:** 2014-03-25

**Authors:** Chingakham R. Singh, Scott Lovell, Nurjahan Mehzabeen, Wasimul Q. Chowdhury, Eric S. Geanes, Kevin P. Battaile, Jeroen Roelofs

**Affiliations:** a Division of Biology, Kansas State University, 338 Ackert Hall, Manhattan, KS 66506, USA; b Protein Structure Laboratory, University of Kansas, Del Shankel Structural Biology Center, Lawrence, KS 66047, USA; c IMCA-CAT Hauptman–Woodward Medical Research Institute, 9700 South Cass Avenue, Building 435A, Argonne, IL 60439, USA

**Keywords:** Nas2, chaperones, proteasome, PDZ domain

## Abstract

The 26S proteasome is a 2.5 MDa protease dedicated to the degradation of ubiquitinated proteins in eukaryotes. The assembly of this complex containing 66 polypeptides is assisted by at least nine proteasome-specific chaperones. One of these, Nas2, binds to the proteasomal AAA-ATPase subunit Rpt5. The PDZ domain of Nas2 binds to the C-terminal tail of Rpt5; however, it does not require the C-terminus of Rpt5 for binding. Here, the 1.15 Å resolution structure of the PDZ domain of Nas2 is reported. This structure will provide a basis for further insights regarding the structure and function of Nas2 in proteasome assembly.

## Abstract 2

The proteasome-assembly chaperone Nas2 binds to the proteasome subunit Rpt5 using its PDZ domain. The structure of the Nas2 PDZ domain has been determined.

## 1. Introduction   

The eukaryotic proteasome consists of a core particle (CP) and a regulatory particle (RP). The CP is a cylindrically shaped structure with the proteolytic active sites on the inner surface of this complex (Groll *et al.*, 1997 ▶). One or both ends can associate with the RP, forming the 26S proteasome. CP assembly and RP assembly are both assisted by proteasome-specific chaperones (Bedford *et al.*, 2010 ▶). The RP-specific chaperones Nas6, Nas2, Hsm3 and Rpn14 are important for the assembly of a hetero-hexameric AAA-ATPase ring formed by the subunits Rpt1 to Rpt6 (Funakoshi *et al.*, 2009 ▶; Kaneko *et al.*, 2009 ▶; Le Tallec *et al.*, 2009 ▶; Roelofs *et al.*, 2009 ▶; Saeki *et al.*, 2009 ▶). Interestingly, each chaperone largely consists of protein–protein interaction motifs: Nas6 contains seven ankyrin repeats, Hsm3 is formed by armadillo/heat-like repeats, Rpn14 contains a WD40 domain and Nas2 has a predicted PDZ domain. Although structurally different, each chaperone binds to the C-domain of a specific Rpt protein. Structural studies looking at the interaction between Nas6 and Rpt3 (Nakamura *et al.*, 2007 ▶; Roelofs *et al.*, 2009 ▶), Hsm3 and Rpt1 (Barrault *et al.*, 2012 ▶; Takagi *et al.*, 2012 ▶; Park *et al.*, 2013 ▶), and Rpn14 and Rpt6 (Ehlinger *et al.*, 2013 ▶) have provided substantial structural insights. However, no structural data for Nas2, or the human ortholog p27/PSMD9, have been published to date. Binding studies between Nas2 and Rpt5 have shown that deletion of the C-terminal residue of Rpt5 did not impact Nas2 binding (Lee *et al.*, 2011 ▶). Deletion of the last three amino acids, on the other hand, caused a strong reduction in binding. This indicates that the tail of Rpt5 is important for binding with Nas2 (Lee *et al.*, 2011 ▶). This same tail is also involved in the association between the Rpt5 subunit and the CP (Tian *et al.*, 2011 ▶; Beck *et al.*, 2012 ▶; Lander *et al.*, 2012 ▶), which suggests that the binding of Nas2 prevents the association of Rpt5, or Rpt5-containing complexes, with the CP. The structure of the Nas2 PDZ domain reported here is the first step towards a structural understanding of this interaction.

## 2. Materials and methods   

### 2.1. Macromolecule production   

The open reading frame of *Saccharomyces cerevisiae* Nas2 was subcloned into a pGEX-6P1-derived plasmid, creating plasmid pJR500 (Lee *et al.*, 2011 ▶). Based on domain and secondary-structure prediction analyses, two truncated versions of Nas2 containing the single PDZ domain were generated: Nas2LND, covering amino acids Gln126–Leu220 (pJR607), and Nas2ND, covering Asn91–Leu220 (pJR606). Expression and purification of the different proteins were conducted as described previously for Hsm3 (Park *et al.*, 2013 ▶). Basically, *Escherichia coli* cells induced to express the GST-fused proteins were lysed. The fusion proteins were bound to glutathione resin. The affinity tag was removed by incubation with PreScission protease and the eluted proteins were further purified by size-exclusion chromatography using a Superdex 200 (10/300 GL) column.

### 2.2. Crystallization   

A purified sample of Nas2LND (Table 1 ▶) concentrated to 7.0 mg ml^−1^ in 50 m*M* NaCl, 50 m*M* Tris–HCl pH 6.8, 1 m*M* DTT, 1 m*M* EDTA was used for crystallization screening. All crystallization experiments were conducted by sitting-drop vapor diffusion in Compact Jr plates (Emerald Bio) using equal volumes of protein and crystallization solutions equilibrated against 75 µl of the latter. After two months, prismatic crystals of ∼0.2 mm in length were obtained at 4°C from Wizard Classic 4 screen (Emerald Bio) condition No. 26 [10%(*w*/*v*) PEG 2000 MME, 100 m*M* sodium acetate/acetic acid pH 5.5, 200 m*M* ammonium sulfate].

### 2.3. Data collection and processing   

Crystal samples were transferred to a fresh drop composed of 75% crystallization solution and 25%(*v*/*v*) PEG 400 and stored in liquid nitrogen. Diffraction data were collected in-house using a Rigaku RU-H3R rotating-anode generator (Cu *K*α) equipped with Osmic Blue confocal mirrors and an R-AXIS IV^++^ image-plate detector. High-resolution data were collected on beamline 17-ID at the Advanced Photon Source using a Dectris Pilatus 6M pixel-array detector. Intensities were integrated using *XDS* (Kabsch, 2010*a* ▶,*b* ▶) and Laue class analysis and data scaling was performed with *AIMLESS* (Evans, 2011 ▶; Evans & Murshudov, 2013 ▶), which indicated that the highest probability Laue class was 4/*mmm* and the most likely space groups were *P*4_1_2_1_2 or *P*4_3_2_1_2. Data-collection statistics are given in Table 2 ▶.

### 2.4. Structure solution and refinement   

The Matthews coefficient (Matthews, 1968 ▶) indicated that the asymmetric unit contained a single molecule (*V*_M_ = 1.99 Å^3^ Da^−1^, ∼40% solvent). Structure solution was conducted by molecular replacement with *BALBES* (Long *et al.*, 2008 ▶), in which the top solution was obtained using the GRASP55 GRASP domain (PDB entry 3rle; Truschel *et al.*, 2011 ▶) as the search model. Following initial refinement with *PHENIX* (Adams *et al.*, 2010 ▶) the *R* and *R*_free_ converged at 42.8 and 46.4%, respectively. The model was improved by automated model building with *ARP*/*wARP* (Langer *et al.*, 2008 ▶) and the *R* and *R*_free_ converged at 20.7 and 23.5%, respectively, following refinement of this model. To check that the correct space group had been assigned, the model was used for a molecular-replacement search with *Phaser* (McCoy *et al.*, 2007 ▶) checking all space groups with 422 point symmetry. The top solution was obtained in space group *P*4_1_2_1_2 (LLG = 13 591) which was used from this point forward. Additional structure refinement and manual model building with the high-resolution synchrotron data were conducted with *PHENIX* and *Coot* (Emsley *et al.*, 2010 ▶), respectively. All atoms were refined with anisotropic atomic displacement parameters. H atoms were placed in idealized positions and refined with a riding model. Structure validation was conducted with *MolProbity* (Chen *et al.*, 2010 ▶) and figures were prepared using the *CCP*4*mg* package (McNicholas *et al.*, 2011 ▶). Structure-refinement statistics are given in Table 2 ▶.

## 3. Results and discussion   

### 3.1. Structure of the Nas2 PDZ domain   

**Figure 1:**
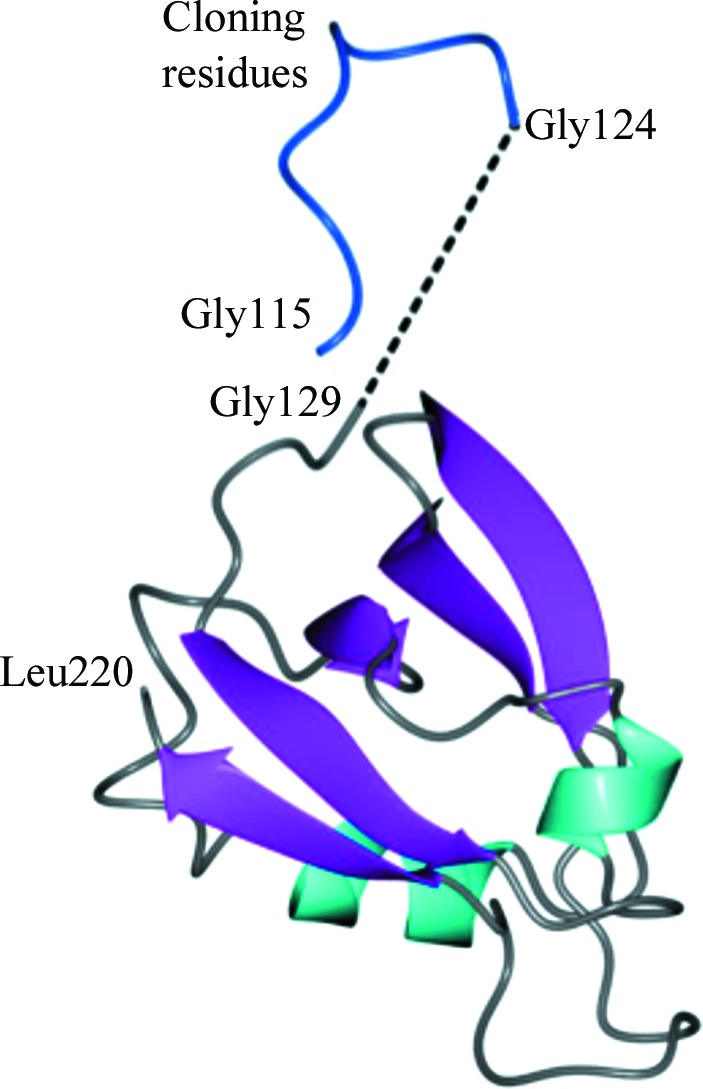
Structure of the Nas2 PDZ domain. Asymmetric unit of Nas2LND colored by secondary structure: sheet, magenta; helix, cyan. The disordered region is indicated by the dashed line. N-terminal residues resulting from cloning are colored blue.

The C-terminal half of Nas2 constitutes a PDZ domain, while the N-terminal half has no predicted domain structure (Supplementary Fig. S1*a* 1). Nas2 directly interacts with the proteasomal ATPase subunit Rpt5, requiring the last three amino acids of Rpt5 for binding (Lee *et al.*, 2011 ▶). Since many PDZ domains bind to C-termini (Lee & Zheng, 2010 ▶), this suggests that the Nas2 PDZ domain is responsible for the interaction with Rpt5. Surprisingly, however, deletion of only the ultimate C-terminal residue of Rpt5 had little impact on Rpt5–Nas2 binding (Lee *et al.*, 2011 ▶). To gain a structural understanding of the interaction between Nas2 and Rpt5, we attempted to crystallize or co-crystallize Nas2 with the C-domain of Rpt5. Since no crystals were obtained, we created two truncated forms of Nas2 with the goal of minimizing the sequence around the PDZ domain. The truncated forms of Nas2 retained their ability to bind to the Rpt5 C-­domain and showed only a modest reduction in affinity as measured using bio-layer interferometry (Supplementary Fig. S1*b*). Nas2LND produced well diffracting crystals after two months, enabling us to determine a structure comprised of amino acids Gln129–Leu220 in the asymmetric unit (Fig. 1 ▶). Nas2LND adopts a fold consisting of five β-sheets and two α-helices commonly found amongst PDZ-domain structures (Fig. 1 ▶). N-terminal residues from cloning that spanned Gly115–Gly124 could be traced in the electron-density maps (blue in Fig. 1 ▶). However, residues between Gly124 and Gly129 were disordered and could not be modeled. Fig. 1 ▶ shows the N-­terminal fragment Gly115–Gly124 belonging to the same chain; however, we cannot exclude the possibility it is derived from a neighboring molecule in the crystal (Supplementary Fig. S2). Following refinement, recurring electron density was observed in two regions. The first was modeled as a disordered PEG 400 fragment obtained from the cryoprotectant (Supplementary Fig. S3*a*). The second region was observed between Lys119 and Lys120 and was assigned as a sulfate ion (Supplementary Fig. S3*b*).

### 3.2. Structural comparison with other PDZ domains   

**Figure 2:**
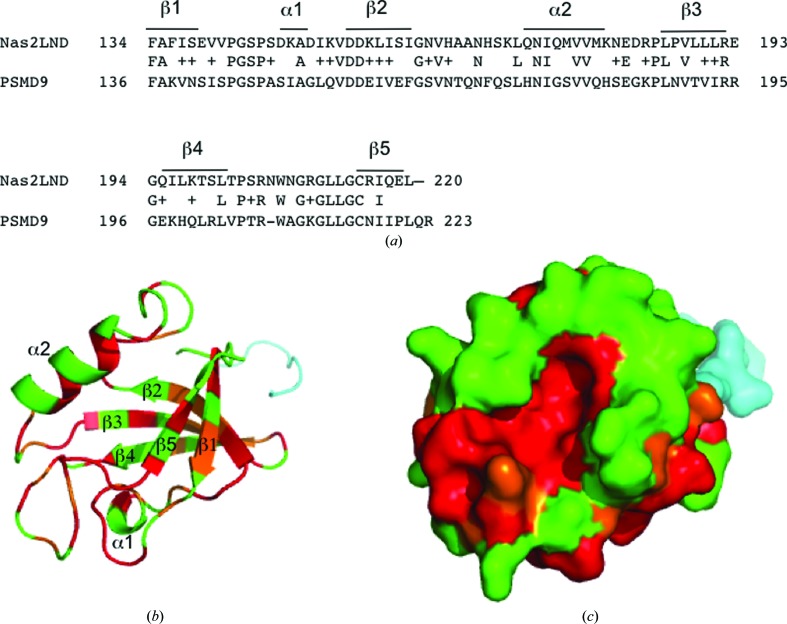
Mapping residues that are conserved between Nas2 and the human ortholog PSMD9. (*a*) Alignment of Nas2 PDZ-domain residues with the human ortholog PSMD9. Residues modeled in the crystal structure of Nas2 were used in a *BLASTp* search against the human protein RefSeq database. The top hit was the human ortholog of Nas2, PSMD9, showing 42% identity and 64% conserved residues (the latter are indicated with +). (*b*) Graphic structure of Nas2 in green. Based on an alignment between Nas2 and PSMD9, conserved residues were colored orange and identical residues red. The cloning-derived residues are in blue. (*c*) Surface representation of (*b*). Note the conservation in the groove of the PDZ domain between α-helix 2 and β-strand 5. In PDZ-domain proteins this region commonly binds peptides/C-termini from their binding partner.

**Figure 3:**
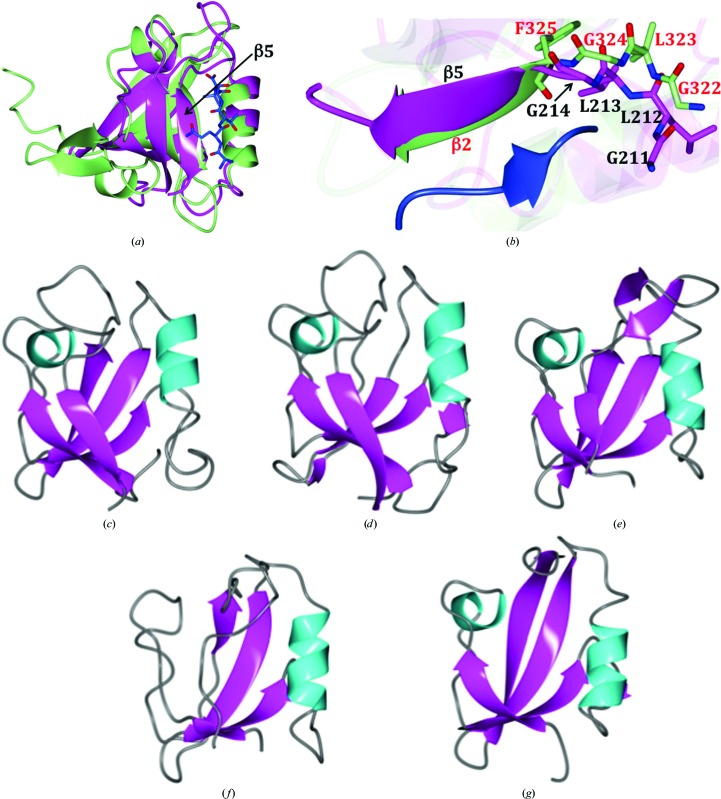
(*a*) Superposition of Nas2LND (magenta) with a canonical PDZ domain (PDB entry 1be9, green). The peptide ligand for 1be9 is shown as blue cylinders. β-Sheet 5 of Nas2LND is indicated. Superposition of a canonical PDZ domain (PDB entry 1be9) with Nas2LND was conducted using *GESAMT* (Krissinel, 2012 ▶), which yielded an r.m.s.d. of 2.27 Å for 59 aligned residues. (*b*) Enlarged view of the β5/α2 region of Nas2LND superimposed with 1be9. The peptide ligand for 1be9 is shown as a blue ribbon. The GLGF (red) motif of 1be9 and similar residues in front of Nas2LND β-sheet 5, GLLG (black), are indicated. (*c*–*f*) Superposition of specified regions of PDZ-domain homologs onto Nas2LND colored by secondary structure as in Fig. 1 ▶. (*c*) Nas2LND, (*d*) PDB entry 3rle (chain *A*, residues 7–104), (*e*) PDB entry 3id4 (chain *A*, residues 223–307), (*f*) PDB entry 3i1e (chain *B*, residues 3–85) and (*g*) PDB entry 3gdv (chain *C*, residues 279–410).

The structured residues of Nas2 were aligned with the human ortholog p27/PSMD9 (Fig. 2 ▶ *a*). Several conserved residues are clustered on the surface of the PDZ domain (Figs. 2 ▶ *b* and 2 ▶ *c*). Interestingly, there is a group of identical hydrophobic residues covering the region between α-helix 2 and β-sheet 5 (Figs. 2 ▶ *b* and 2 ▶ *c*), a region that is known to be the peptide-binding groove in other PDZ domains (Lee & Zheng, 2010 ▶; Fig. 3 ▶ *a*). Similar to what was reported for GRASP55 (PDB entry 3rle), the groove was formed by the final, instead of the second, β-strand (Figs. 3 ▶ *a* and 3 ▶ *b*), indicating a similar circular permutation from a typical eukaryotic PDZ domain (Truschel *et al.*, 2011 ▶). At the entrance of this groove there are conserved charged residues. Besides the groove, there are other surface areas that appear to contain a cluster of conserved residues, maybe facilitating additional binding interactions as the residues do not appear to be structural.

To compare the structure of Nas2 PDZ with a typical PDZ domain that utilizes a GLGF motif for binding the C-terminus of other proteins (Lee & Zheng, 2010 ▶), we aligned the structure with PDB entry 1be9 (Doyle *et al.*, 1996 ▶). The GLGF motif is located before the β-strand 2 of 1be9. This compares to the region in front of β-strand 5 in Nas2LND owing to the altered β-strand arrangement (Figs. 3 ▶ *a* and 3 ▶ *b*). Interestingly, a similar motif is found in this region in Nas2LND (GLLG; Figs. 2 ▶ *a* and 3 ▶ *b*). Next, the structure of Nas2LND was also compared with the top structures obtained from a *DALI* search (Holm & Rosenström, 2010 ▶), and the coordinates were superimposed using *GESAMT* (Krissinel, 2012 ▶). Overall, the structure of Nas2LND is similar to these homologous structures as indicated from superpositions: r.m.s.d.s of 1.30 Å with PDB entry 3rle (chain *A*, residues 7–­104; 87 residues; Truschel *et al.*, 2011 ▶), 1.30 Å with PDB entry 3id4 (chain *A*, residues 223–307; 74 residues; Li *et al.*, 2009 ▶), 1.68 Å with PDB entry 3i1e (chain *B*, residues 3–85; 75 residues; Northeast Structural Genomics Consortium, unpublished work) and 1.68 Å with PDB entry 3gdv (chain *C*, residues 279–410; 72 residues; Sohn *et al.*, 2009 ▶). A side-by-side comparison of these structures is shown in Figs. 3 ▶(*c*)–3 ▶(*f*).

## 4. Conclusion   

The 1.15 Å resolution crystal structure of the C-terminal region of Nas2 shows it contains a PDZ domain. Nas2 lacks the GLGF motif commonly found just prior to β-strand 2 in PDZ domains. This motif interacts with the carboxy-termini of PDZ-binding partners (Lee & Zheng, 2010 ▶). The absence is not surprising as the Nas2 structure shows that β-strand 5, instead of the common β-strand 2, contributes to the putative peptide-binding groove, causing a different loop arrangement. In front of β-strand 5 a similar sequence is present (GLLG). However, the role of this sequence in interactions with C-­termini of Nas2-binding partners remains to be determined. In particular, the binding between Nas2 and Rpt5 does not appear to be affected by deletion of the Rpt5 C-terminal residue (Lee *et al.*, 2011 ▶). The reported Nas2 structure will provide the basis for further insights regarding the structure and function of Nas2 in proteasome assembly, as it will facilitate molecular docking of the tail of Rpt5 as well as enabling the design of Nas2 mutants based on the putative binding region.

## Supplementary Material

PDB reference: proteasome-assembly chaperone Nas2 PDZ domain, 4o06

Supplementary figures. DOI: 10.1107/S2053230X14003884/hv5251sup1.pdf

## Figures and Tables

**Table 1:** Amino-acid sequence of the Nas2LND fragment that crystallized

Source organism	*S. cerevisiae* strain ATCC 204508/S288c
DNA source	*S. cerevisiae* strain ATCC 204508/S288c
Expression vector	pGEX-6P1-derived plasmid
Expression host	*E. coli* BL21(DE3)
Complete amino-acid sequence of the construct produced	GPLTRRASVGSQAIQYTIPFAFISEVVPGSPSDKADIKVDDKLISIGNVHAANHSKLQNIQMVVMKNEDRPLPVLLLREGQILKTSLTPSRNWNGRGLLGCRIQEL
Vector-derived residues	GPLTRRASVGS

**Table 2:** Data-collection statistics for Nas2LND refined to 1.15 Å resolution Values in parentheses are for the highest resolution shell.

Unit-cell parameters (Å, °)	*a* = 39.97, *b* = 39.97 *c* = 115.80, α = β = γ = 90
Space group	*P*4_1_2_1_2
Resolution (Å)	39.97–1.15 (1.17–1.15)
Wavelength (Å)	1.0000
Temperature (°C)	−173
Observed reflections	808924
Unique reflections	33545
〈*I*/σ(*I*)〉	23.0 (3.4)
Completeness (%)	97.9 (95.1)
Multiplicity	24.1 (17.6)
*R*_merge_ † (%)	7.7 (107.0)
*R*_meas_ ‡ (%)	7.8 (110.0)
*R*_p.i.m._ ‡ (%)	1.6 (25.6)
CC_1/2_ §	0.999 (0.880)

† *R*_merge_ = 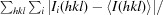 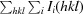, where *I_i_*(*hkl*) is the intensity measured for the *i*th reflection and 〈*I*(*hkl*)〉 is the average intensity of all reflections with indices *hkl*.

‡ *R*_meas_ is the redundancy-independent (multiplicity-weighted) *R*_merge_ (Evans, 2006 ▶, 2011 ▶). *R*_p.i.m._ is the precision-indicating (multiplicity-weighted) *R*_merge_ (Diederichs & Karplus, 1997 ▶; Weiss, 2001 ▶).

§ CC_1/2_ is the correlation coefficient of the mean intensities between two random half-sets of data (Karplus & Diederichs, 2012 ▶; Evans, 2012 ▶).

**Table 3:** Structure refinement

Refinement	
Resolution (Å)	32.90–1.15
Reflections (working/test)	31796/1698
*R* factor/*R*_free_ † (%)	17.2/19.4
No. of atoms (protein/sulfate/PEG/water)	804/5/8/98
Model quality	
R.m.s. deviations	
Bond lengths (Å)	0.012
Bond angles (°)	1.356
Average *B* factor (Å^2^)	
All atoms	18.0
Protein	16.4
Sulfate	30.6
PEG	29.5
Water	28.8
Coordinate error (maximum likelihood) (Å)	0.12
Ramachandran plot	
Most favored (%)	96.2
Additionally allowed (%)	3.8

† *R* factor = 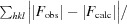 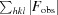
Σ*_hkl_*; *R*_free_ is calculated in an identical manner using 5% of randomly selected reflections that were not included in the refinement.

## Footnotes

1 Supporting information has been deposited in the IUCr electronic archive (Reference: HV5251).

## References

[bb1] Adams, P. D. *et al.* (2010). *Acta Cryst.* D**66**, 213–221.

[bb2] Barrault, M.-B., Richet, N., Godard, C., Murciano, B., Le Tallec, B., Rousseau, E., Legrand, P., Charbonnier, J.-B., Le Du, M.-H., Guérois, R., Ochsenbein, F. & Peyroche, A. (2012). *Proc. Natl Acad. Sci. USA*, **109**, E1001–E1010. 10.1073/pnas.1116538109 PMC3340050 22460800

[bb3] Beck, F., Unverdorben, P., Bohn, S., Schweitzer, A., Pfeifer, G., Sakata, E., Nickell, S., Plitzko, J. M., Villa, E., Baumeister, W. & Förster, F. (2012). *Proc. Natl Acad. Sci. USA*, **109**, 14870–14875. 10.1073/pnas.1213333109 PMC3443124 22927375

[bb4] Bedford, L., Paine, S., Sheppard, P. W., Mayer, R. J. & Roelofs, J. (2010). *Trends Cell Biol.* **20**, 391–401. 10.1016/j.tcb.2010.03.007 PMC2902798 20427185

[bb5] Chen, V. B., Arendall, W. B., Headd, J. J., Keedy, D. A., Immormino, R. M., Kapral, G. J., Murray, L. W., Richardson, J. S. & Richardson, D. C. (2010). *Acta Cryst.* D**66**, 12–21. 10.1107/S0907444909042073 PMC2803126 20057044

[bb6] Diederichs, K. & Karplus, P. A. (1997). *Nature Struct. Biol.* **4**, 269–275. 10.1038/nsb0497-269 9095194

[bb7] Doyle, D. A., Lee, A., Lewis, J., Kim, E., Sheng, M. & MacKinnon, R. (1996). *Cell*, **85**, 1067–1076. 10.1016/s0092-8674(00)81307-0 8674113

[bb8] Ehlinger, A., Park, S., Fahmy, A., Lary, J. W., Cole, J. L., Finley, D. & Walters, K. J. (2013). *Structure*, **21**, 753–765. 10.1016/j.str.2013.02.021 PMC3670613 23562395

[bb9] Emsley, P., Lohkamp, B., Scott, W. G. & Cowtan, K. (2010). *Acta Cryst.* D**66**, 486–501. 10.1107/S0907444910007493 PMC2852313 20383002

[bb10] Evans, P. (2006). *Acta Cryst.* D**62**, 72–82. 10.1107/S0907444905036693 16369096

[bb11] Evans, P. R. (2011). *Acta Cryst.* D**67**, 282–292. 10.1107/S090744491003982X PMC3069743 21460446

[bb12] Evans, P. (2012). *Science*, **336**, 986–987. 10.1126/science.1222162 22628641

[bb13] Evans, P. R. & Murshudov, G. N. (2013). *Acta Cryst.* D**69**, 1204–1214. 10.1107/S0907444913000061 PMC3689523 23793146

[bb14] Funakoshi, M., Tomko, R. J. Jr, Kobayashi, H. & Hochstrasser, M. (2009). *Cell*, **137**, 887–899. 10.1016/j.cell.2009.04.061 PMC2718848 19446322

[bb15] Groll, M., Ditzel, L., Löwe, J., Stock, D., Bochtler, M., Bartunik, H. D. & Huber, R. (1997). *Nature (London)*, **386**, 463–471. 10.1038/386463a0 9087403

[bb16] Holm, L. & Rosenström, P. (2010). *Nucleic Acids Res.* **38**, W545–W549. 10.1093/nar/gkq366 PMC2896194 20457744

[bb17] Kabsch, W. (2010*a*). *Acta Cryst.* D**66**, 125–132. 10.1107/S0907444909047337 PMC2815665 20124692

[bb18] Kabsch, W. (2010*b*). *Acta Cryst.* D**66**, 133–144. 10.1107/S0907444909047374 PMC2815666 20124693

[bb19] Kaneko, T., Hamazaki, J., Iemura, S., Sasaki, K., Furuyama, K., Natsume, T., Tanaka, K. & Murata, S. (2009). *Cell*, **137**, 914–925. 10.1016/j.cell.2009.05.008 19490896

[bb20] Karplus, P. A. & Diederichs, K. (2012). *Science*, **336**, 1030–1033. 10.1126/science.1218231 PMC3457925 22628654

[bb21] Krissinel, E. (2012). *J. Mol. Biochem.* **1**, 76–85. PMC5117261 27882309

[bb22] Lander, G. C., Estrin, E., Matyskiela, M. E., Bashore, C., Nogales, E. & Martin, A. (2012). *Nature (London)*, **482**, 186–191. 10.1038/nature10774 PMC3285539 22237024

[bb23] Langer, G., Cohen, S. X., Lamzin, V. S. & Perrakis, A. (2008). *Nature Protoc.* **3**, 1171–1179. 10.1038/nprot.2008.91 PMC2582149 18600222

[bb25] Lee, H.-J. & Zheng, J. J. (2010). *Cell Commun. Signal.* **8**, 8. 10.1186/1478-811X-8-8 PMC2891790 20509869

[bb24] Lee, S. Y.-C., De La Mota-Peynado, A. & Roelofs, J. (2011). *J. Biol. Chem.* **286**, 36641–36651. 10.1074/jbc.M111.280875 PMC3196109 21878651

[bb26] Le Tallec, B., Barrault, M.-B., Guérois, R., Carré, T. & Peyroche, A. (2009). *Mol. Cell*, **33**, 389–399. 10.1016/j.molcel.2009.01.010 19217412

[bb27] Li, X., Wang, B., Feng, L., Kang, H., Qi, Y., Wang, J. & Shi, Y. (2009). *Proc. Natl Acad. Sci. USA*, **106**, 14837–14842. 10.1073/pnas.0903289106 PMC2736447 19706448

[bb28] Long, F., Vagin, A. A., Young, P. & Murshudov, G. N. (2008). *Acta Cryst.* D**64**, 125–132. 10.1107/S0907444907050172 PMC2394813 18094476

[bb29] Matthews, B. W. (1968). *J. Mol. Biol.* **33**, 491–497. 10.1016/0022-2836(68)90205-2 5700707

[bb30] McCoy, A. J., Grosse-Kunstleve, R. W., Adams, P. D., Winn, M. D., Storoni, L. C. & Read, R. J. (2007). *J. Appl. Cryst.* **40**, 658–674. 10.1107/S0021889807021206 PMC2483472 19461840

[bb31] McNicholas, S., Potterton, E., Wilson, K. S. & Noble, M. E. M. (2011). *Acta Cryst.* D**67**, 386–394. 10.1107/S0907444911007281 PMC3069754 21460457

[bb32] Nakamura, Y., Umehara, T., Tanaka, A., Horikoshi, M., Padmanabhan, B. & Yokoyama, S. (2007). *Biochem. Biophys. Res. Commun.* **359**, 503–509. 10.1016/j.bbrc.2007.05.138 17555716

[bb33] Park, S., Li, X., Kim, H. M., Singh, C. R., Tian, G., Hoyt, M. A., Lovell, S., Battaile, K. P., Zolkiewski, M., Coffino, P., Roelofs, J., Cheng, Y. & Finley, D. (2013). *Nature (London)*, **497**, 512–516. 10.1038/nature12123 PMC3687086 23644457

[bb34] Roelofs, J., Park, S., Haas, W., Tian, G., McAllister, F. E., Huo, Y., Lee, B.-H., Zhang, F., Shi, Y., Gygi, S. P. & Finley, D. (2009). *Nature (London)*, **459**, 861–865. 10.1038/nature08063 PMC2727592 19412159

[bb35] Saeki, Y., Toh-e, A., Kudo, T., Kawamura, H. & Tanaka, K. (2009). *Cell*, **137**, 900–913. 10.1016/j.cell.2009.05.005 19446323

[bb36] Sohn, J., Grant, R. A. & Sauer, R. T. (2009). *Structure*, **17**, 1411–1421. 10.1016/j.str.2009.07.017 PMC2764547 19836340

[bb37] Takagi, K., Kim, S., Yukii, H., Ueno, M., Morishita, R., Endo, Y., Kato, K., Tanaka, K., Saeki, Y. & Mizushima, T. (2012). *J. Biol. Chem.* **287**, 12172–12182. 10.1074/jbc.M112.345876 PMC3320968 22334676

[bb38] Tian, G., Park, S., Lee, M. J., Huck, B., McAllister, F., Hill, C. P., Gygi, S. P. & Finley, D. (2011). *Nature Struct. Mol. Biol.* **18**, 1259–1267. 10.1038/nsmb.2147 PMC3210322 22037170

[bb39] Truschel, S. T., Sengupta, D., Foote, A., Heroux, A., Macbeth, M. R. & Linstedt, A. D. (2011). *J. Biol. Chem.* **286**, 20125–20129. 10.1074/jbc.C111.245324 PMC3121478 21515684

[bb40] Weiss, M. S. (2001). *J. Appl. Cryst.* **34**, 130–135.

