# Supplementary material for: 1.15 Å resolution structure of the proteasome-assembly chaperone Nas2 PDZ domain
Source: Acta Crystallogr F Struct Biol Commun. 2014 Mar 25;70(Pt 4):418–23. doi: 10.1107/S2053230X14003884 (PMC3976055; doi:10.1107/S2053230X14003884)
Supplement: Supplementary file 1 [file f-70-00418-sup1.pdf]

1.15 Å Resolution Structure of the Proteasome Assembly Chaperone Nas2 PDZ Domain

Chingakham R. Singh[a], Scott Lovell[b], Nurjahan Mehzabeen[b], Wasimul Q. Chowdhury[a], Eric Geanes[a], Kevin P. Battaile[c] and Jeroen Roelofs[a]*

[a]Division of Biology, Kansas State University, 338 Ackert Hall, Manhattan, KS, 66506, United States
[b]Protein Structure Laboratory, University of Kansas, Del Shankel Structural Biology Center, Lawrence, KS, 66047, United States
[c] IMCA-CAT Hauptman-Woodward Medical Research Institute, 9700 South Cass Avenue, Building 435A, Argonne, IL, 60439, United States

Correspondence email: jroelofs@ksu.edu

Supplementary figures

A

B

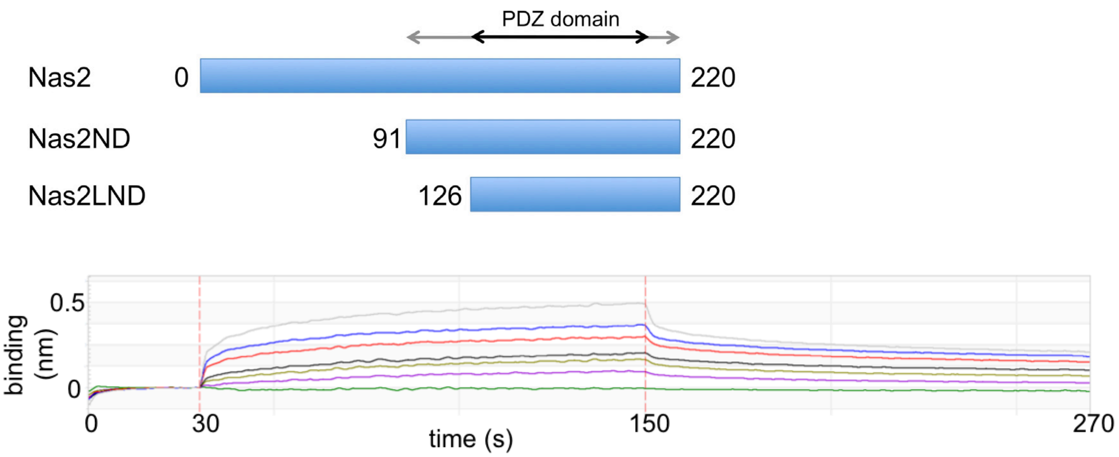

bait	analyte	K_D	k_{on} ($M^{-1}s^{-1}$)	k_{off} (s^{-1})
H_6-$Rpt5^C$	Nas2 (25 kDa)	1.4 μM	9.5×10^3	1.3×10^{-2}
H_6-$Rpt5^C$	Nas2LN (15 kDa)	3.4 μM	7.6×10^4	2.6×10^{-1}
H_6-$Rpt5^C$	Nas2LND (11 kDa)	8.5 μM	4.6×10^4	3.9×10^{-1}

Fig. S1. The PDZ domain of Nas2 binds Rpt5.
(**A**) Topology of Nas2 and truncations used in this study. Predicted region of PDZ domain is indicated. Both truncations bind to the C-domain of Rpt5 ($Rpt5^C$) as determined by GST-pulldown assays using assays described previously (data not shown; Lee *et al*., 2011). (**B**) Quantitative analysis of Nas2-Rpt5 C-domain interaction using Bio-Layer Interferometry on the BLItz (ForteBio). The C-domain of Rpt5 (Lee *et al*., 2011) was captured on Ni-NTA biosensor (ForteBio), and binding with increasing concentrations of analyte was measured using the BLItz. Upper panel shows base-line corrected data for the binding of Rpt5 C-domain with Nas2. Table shows quantitative analyses for Nas2 and the truncated versions Nas2ND and Nas2LND.

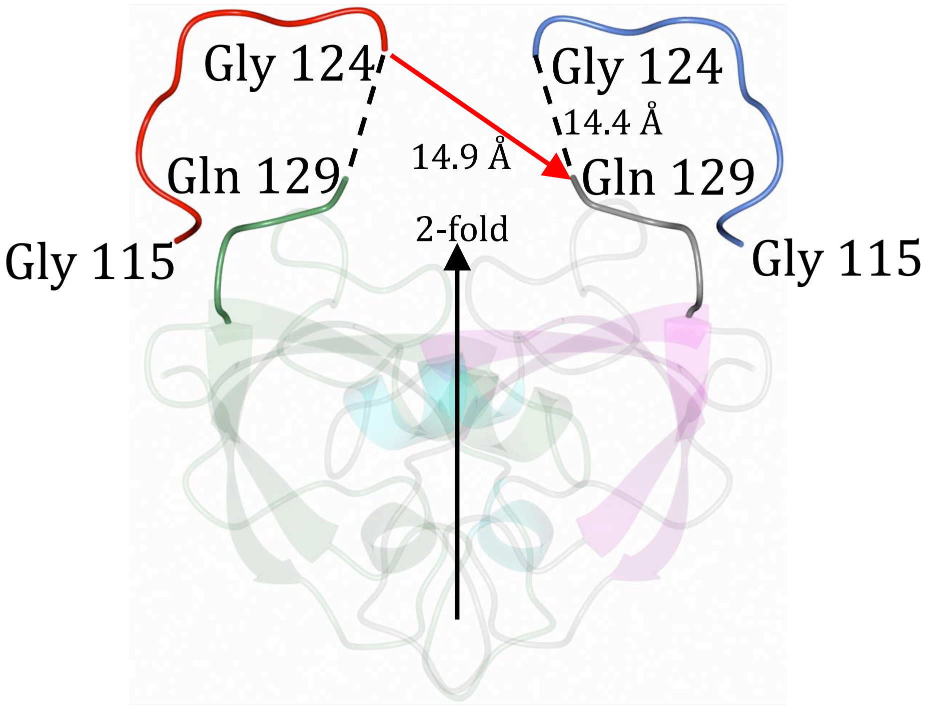

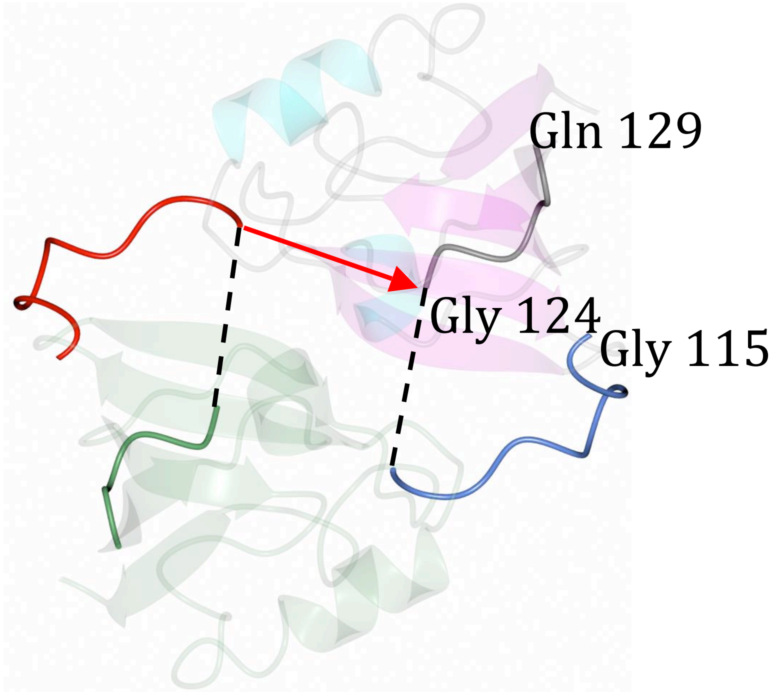

Figure S2. **Top**: Asymmetric unit of Nas2 LND colored by secondary structure: sheet (magenta) and helix (cyan). The disordered region is indicated by the dashed line. N-terminal residues resulting from cloning that could be modeled are colored blue. A molecule related by a 2-fold crystallographic axis (y, x –z) is colored green and its respective N-terminal residues resulting from cloning are colored red. View is normal to the crystallographic 2-fold axis. The distance between Cα atoms of Gly 124 and Gln 129 is 14.4 Å in the current asymmetric unit. However, a comparable distance is found between Cα atoms of Gly 124 from a molecule related by a crystallographic 2-fold axis and Gln 129 in the asymmetric unit is comparable 14.9 Å. Therefore it is not entirely clear if the N-terminal residues are connected to Gln 129 as modeled in the current asymmetric unit or as indicated by the red arrow. However, the orientation of Gly124 and Gln129 in the asymmetric unit appears to be sensible. **Bottom:** Same as the top figure but viewed along the crystallographic 2-fold axis.

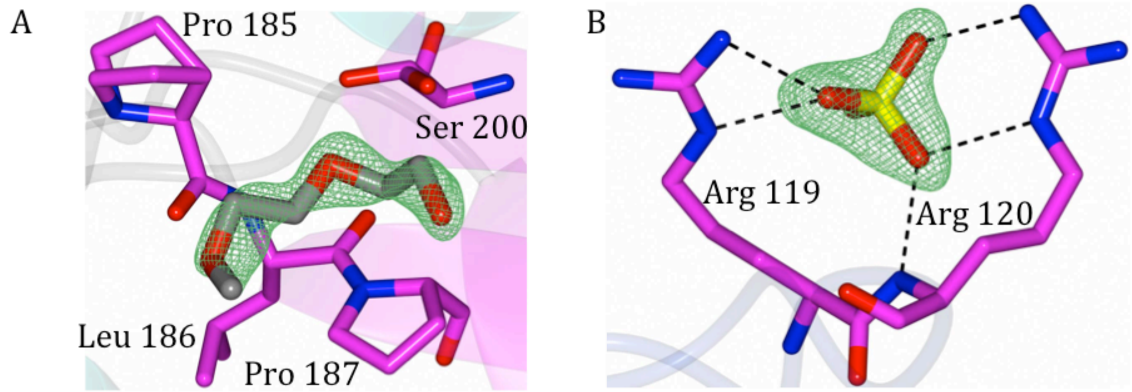

Fig. S3. Structural detail of Nas2 PDZ domain.
(A) Binding region and Fo-Fc electron density map (green mesh) contoured at 3σ for the disordered PEG 400 fragment. Pro 185 and Ser 200 were modeled in alternate conformations as shown. **(B)** Fo-Fc electron density map (green mesh) contoured at 3σ for the modeled sulfate ion coordinated between Arg 119 and Arg 120.

```
                          :         :         :         :         |         :         :         :         :        100
0001 Nas2LND GPLTRRASVQYTIPFAFISEVVPGSPSDKADIKVDDKLISIGNVHAANHSKLQNIQMVVMKNEDRPLPVLLLREGQILKTSLTPSRNWNGRGLLGCRIQEL
0002 3rleA   ---------PGGGEGYHVLRVQENSPGHRAGLEPFDFIVSINSRLNK---DNDTLKDLLKANVEKPVKXLIYSSLELRETSVTPSNLWGGQGLLGVSIRFC
0003 3id4A   --------------EPVLENVQPNSAASKAGLQAGDRIVKVDGQPLT---QWVTFVMLVRDNPGKSLALEIERQGSPLSLTLIPESK-----FVGIEPK-
0004 3i1eB   --------------GVYVLSVKEDVPAAG-ILHAGDLITEIDGQS---FKSSQEFIDYIHSKVGDTVKIKYKHGNKNEEASIKLTAIDK---GIGILEHH-
0005 3gdvA   -------------QGIVVNEVSPDGPAANAGIQVNDLIISVDNKP-----SALETMAQVAEIPGSVIPVVVMRDDKQLTLQVTIQEYP-------------

                          :         :         :         :         |         :         :         :         :        100
0001 Nas2LND LLLLLLLLLLLLLLLEEEEEELLLLHHHHLLLLLLLEEEEELLLLLLLLLLHHHHHHHHHLLLLLEEEEEEELLEEEEEEELLLLLLLLLLLLLEEEEEEL
0002 3rleA   ---------LLLLEEEEEEEELLLLHHHHLLLLLLEEEEEELEELLL---LLLHHHHHHHHLLLLLEEEEEEELLLEEEEEELLLLLLLLLLLLLEEEEEE
0003

 3id4A   --------------LLLEEEELLLLHHHHLLLLLLLLEEEEEELLLLLL---LHHHHHHHHHLLLLLLEEEEEEEELLEEEEEEEELLEEE-----ELLEEEL-
0004 3i1eB   --------------LEEEEEEELLLLHHHL-LLLLLLEELEELLEE---LLLHHHHHHHHHHHLLLLEEEEELEELLEELLEEEELEELLL---ELLEEELL-
0005 3gdvA   -------------LLEEEEEEELLLLHHHHHLLLLLLEEEEEELLEE-----LHHHHHHHHHHLLLLEEEEEEEELLEEEEEEEELEELL-------------
```

Fig. S4. Dali analysis of Nas2LND.
Upper panel shows results from a Dali search to identify the closest structural homologs. Lower panel shows secondary structure assignments by DSSP (H/h: helix, E/e: strand, L/l: coil). The most frequent amino acid type is colored in each column.
